# Cancer Cachexia Research and Drug Development: Lessons from Failures and the Promise of Immunomodulation

**DOI:** 10.3390/cancers18172738

**Published:** 2026-08-23

**Authors:** Lingbing Zhang, Jeffrey A. Norton

**Affiliations:** 1 Yinuoke Ltd., Changchun 130012, China; 2 Department of Surgery, School of Medicine, Stanford University, Stanford, CA 94305, USA; janorton@stanford.edu

**Keywords:** cancer cachexia, immune dysregulation, systemic inflammation, multimodal therapy, immunomodulation, R-ketorolac

## Abstract

Cancer cachexia is a multifactorial systemic syndrome characterized by progressive muscle loss, with or without adipose tissue depletion, that cannot be reversed by conventional nutritional support. It affects cancer patients and is associated with reduced treatment tolerance, impaired physical function, poor quality of life, and increased mortality. The understanding of cachexia has evolved recently, from the perception of a simple nutritional disorder to a complex immune–metabolic syndrome, based on tumor–host interactions, systemic inflammation, metabolic dysregulation, and multi-organ dysfunction. This review summarizes the progression of cachexia research, highlighting key findings involving inflammatory cytokines, proteolytic pathways, mitochondrial dysfunction, and immune dysregulation. The development of therapeutic strategies is examined, from early nutritional and appetite-stimulating interventions to contemporary targeted therapies, including ghrelin receptor agonists, cytokine inhibitors, and anabolic agents. Despite advances in mechanistic understanding, numerous trials targeting single pathways have failed to produce meaningful functional or survival benefits, underscoring the limitations of reductionist approaches. Emerging evidence supports a paradigm shift toward multimodal, biomarker-guided, and patient-centered interventions that address the interconnected biological mechanisms underlying cachexia. Particular emphasis is given to novel immunomodulatory strategies, including agents such as R-ketorolac, which may restore immune homeostasis and target the root causes of cachexia. It is hypothesized that future therapeutic success will likely depend on integrated approaches combining immunological, metabolic, nutritional, and rehabilitative interventions.

## Simple Summary

Cancer cachexia is a serious condition that affects individuals with cancer, causing an ongoing loss of muscle and occasionally body fat, resulting in weakness, fatigue, reduced quality of life, and poor response to treatment. Unlike ordinary weight loss, cancer cachexia cannot be reversed by eating more or improving nutrition. It is driven by complex interactions among the tumor, the immune system, inflammation, and changes in the body’s metabolism. This review describes the evolution of cancer cachexia and why many treatments targeting a single biological pathway have failed to improve patient outcomes. Current evidence suggests that more effective management will require a combination of approaches, such as medicines, nutritional support, exercise and rehabilitation, tailored to the patient’s condition. Emerging treatments, such as immunomodulatory therapies, restore immune balance and may address the underlying causes of cachexia and offer new therapies for improving treatment tolerance, physical function and survival in patients with cancer.

## 1. Introduction

Cancer cachexia is caused by the presence of certain cancers. It is a widely recognized multifactorial syndrome affecting the whole body. It is characterized by progressive weight loss, skeletal muscle wasting, and metabolic disturbances. It cannot be entirely reversed through standard nutritional support but can be reversed if the patient has a complete anti-tumor response. The syndrome presents with debilitating symptoms such as unintentional weight loss, muscle atrophy, fatigue, anorexia, and generalized weakness, which collectively diminish patient function and overall well-being. These manifestations result from a sustained negative energy balance driven by reduced food intake and heightened metabolic demands related to tumor progression and systemic inflammation [1]. This condition also affects multiple organ systems, altering muscle metabolism, immune responses, and neuroendocrine signaling pathways, thereby contributing to disease progression. As the syndrome advances, patients encounter severe fatigue, apathy, and reduced physical performance, markedly impairing quality of life and complicating clinical management [1,2].

The high prevalence and profound effect on disease outcomes among cancer patients underscore the clinical significance of cancer cachexia. Approximately 50% of individuals with cancer develop cachexia; however, its incidence can vary considerably depending on tumor type and stage, with reported risks ranging from 20% to 80% in cases of advanced malignancies. This syndrome is closely linked to progressive functional decline and decreased survival rates, thereby representing one of the most critical complications associated with the progression of cancer [1,2]. Cachexia has been frequently observed in patients with gastrointestinal and advanced cancers, often manifesting as severe weight loss and muscle wasting prior to diagnosis. These metabolic changes expedite disease progression and exacerbate clinical outcomes by diminishing treatment tolerance and heightening susceptibility to complications. Consequently, the presence of cachexia serves as a significant prognostic indicator in oncological care and underscores the importance of early detection and intervention [1,2].

Furthermore, cancer cachexia substantially contributes to organ dysfunction and physiological decline, chiefly through progressive skeletal muscle depletion and systemic inflammation. These phenomena impair functional capacity, weaken respiratory and cardiac musculature, and disrupt metabolic homeostasis, collectively elevating the risk of organ failure and mortality among cancer patients. Additionally, cachexia often diminishes patients’ tolerance for chemotherapy, radiotherapy, surgery and other anticancer interventions, leading to dose reductions, treatment delays, or cessation of therapy. Clinical implications, including altered treatment outcomes and overall survival rates, underscore the importance of cancer cachexia as a critical therapeutic target in oncology research and clinical practice [2,3]. This review assesses why drug development based on specific pathways in cancer cachexia has been unsuccessful in the past and explores the potential of immunomodulatory therapy for improving treatment tolerance, physical function and patient survival.

A literature search was conducted using PubMed and Google Scholar to identify relevant publications on cancer cachexia and its treatment. The search was restricted to articles published in English, and no lower date limit was applied so as to include all historically significant research alongside recent advances. Search terms included keywords such as “cancer cachexia”, “immunomodulation”, “multimodal therapy”, “drug development”, “immune dysregulation”, and “immunomodulatory agents”, and the reference lists of important articles were screened for additional sources. Publications were selected on the basis of relevance to the pathophysiology, therapeutic evolution, and emerging immunomodulatory approaches for cancer cachexia.

## 2. Historical Overview of Cancer Cachexia Research

Cancer cachexia is frequently observed in patients with advanced malignancies and contributes significantly to morbidity, decreased tolerance to anticancer therapies, reduced quality of life, and increased mortality. Over the past several decades, the scientific understanding of cancer cachexia has evolved from early clinical descriptions of disease-related wasting to a complex mechanistic framework involving metabolic dysregulation, systemic inflammation, and multi-organ interactions. Current research on the syndrome reports advances in experimental biology, immunology, and metabolism, providing clarity on the mechanisms underlying cancer-associated wasting [4,5,6].

### 2.1. Early Recognition of Disease-Associated Wasting

Ancient medical texts mention cancer cachexia, with Hippocratic writings from the fourth century BC describing progressive wasting and weakness in patients with chronic diseases, including cancer. These patients often experienced severe weight loss and poor clinical outcomes, and the condition was hard to reverse despite efforts to provide adequate nutrition. Although the mechanisms behind these observations were not understood then, they signaled early recognition that chronic illness could cause systemic wasting separate from starvation [7].

Throughout the nineteenth and early twentieth centuries, clinicians observed cancer-related wasting. They noted that patients with advanced cancers suffered from significant weight loss, muscle wasting, fatigue, and functional decline. Initially, many attributed this wasting mainly to anorexia and decreased calorie intake. However, experimental tumor models developed in the mid-twentieth century challenged this hypothesis [8].

### 2.2. Experimental Evidence and Tumor–Host Interactions

During the 1950s and early 1960s, experimental studies utilizing tumor-bearing animals provided crucial insights into the systemic nature of cancer cachexia. These studies reported that animals with implanted tumors exhibited weight loss and muscle wasting despite food intake being maintained. Such findings implied that tumor growth could induce metabolic alterations that promote tissue catabolism independent of nutritional deficiency. Further experimental research demonstrated that the injection of tumor cells into experimental animals resulted in a wasting syndrome akin to clinical cachexia. These observations suggested that tumors release circulating factors capable of modifying host metabolism and inducing systemic catabolic responses. The understanding of tumor–host interactions became fundamental to elucidating cachexia pathophysiology, thereby transforming the perspective on nutritional deficiencies [9,10,11,12].

### 2.3. Evolution of Understanding: Metabolic Dysfunction and Energy Imbalance

Across the period from the 1970s to the 1990s, studies showed that cachexia involves intricate metabolic changes rather than straightforward malnutrition. Clinical and experimental evidence indicated that cancer patients suffering from cachexia often had increased resting energy expenditure, disrupted carbohydrate metabolism, and heightened lipid mobilization. These metabolic disruptions led to a negative energy balance, resulting in tissue loss. Additionally, efforts to combat cachexia with intensive nutritional supplementation frequently failed to restore muscle mass or prevent weight loss. These observations underscored the importance of underlying metabolic dysregulation in the development of cachexia [9].

Subsequent research has demonstrated that the loss of skeletal muscle in cachexia is principally caused by the activation of proteolytic pathways, including the ubiquitin–proteasome system and autophagy-related degradation mechanisms. These pathways expedite the degradation of myofibrillar proteins and result in progressive muscle atrophy [13].

Cachexia results in remarkable alterations in adipose tissue metabolism, manifesting as accelerated lipolysis and impaired lipid storage, which leads to rapid depletion of fat mass and contributes to systemic energy imbalance and metabolic dysfunction [14,15]. These metabolic alterations establish cancer cachexia as a systemic syndrome characterized by changes in multiple metabolic pathways rather than a localized effect of tumor growth.

### 2.4. Identification of Inflammatory Mediators

**Figure 1:**
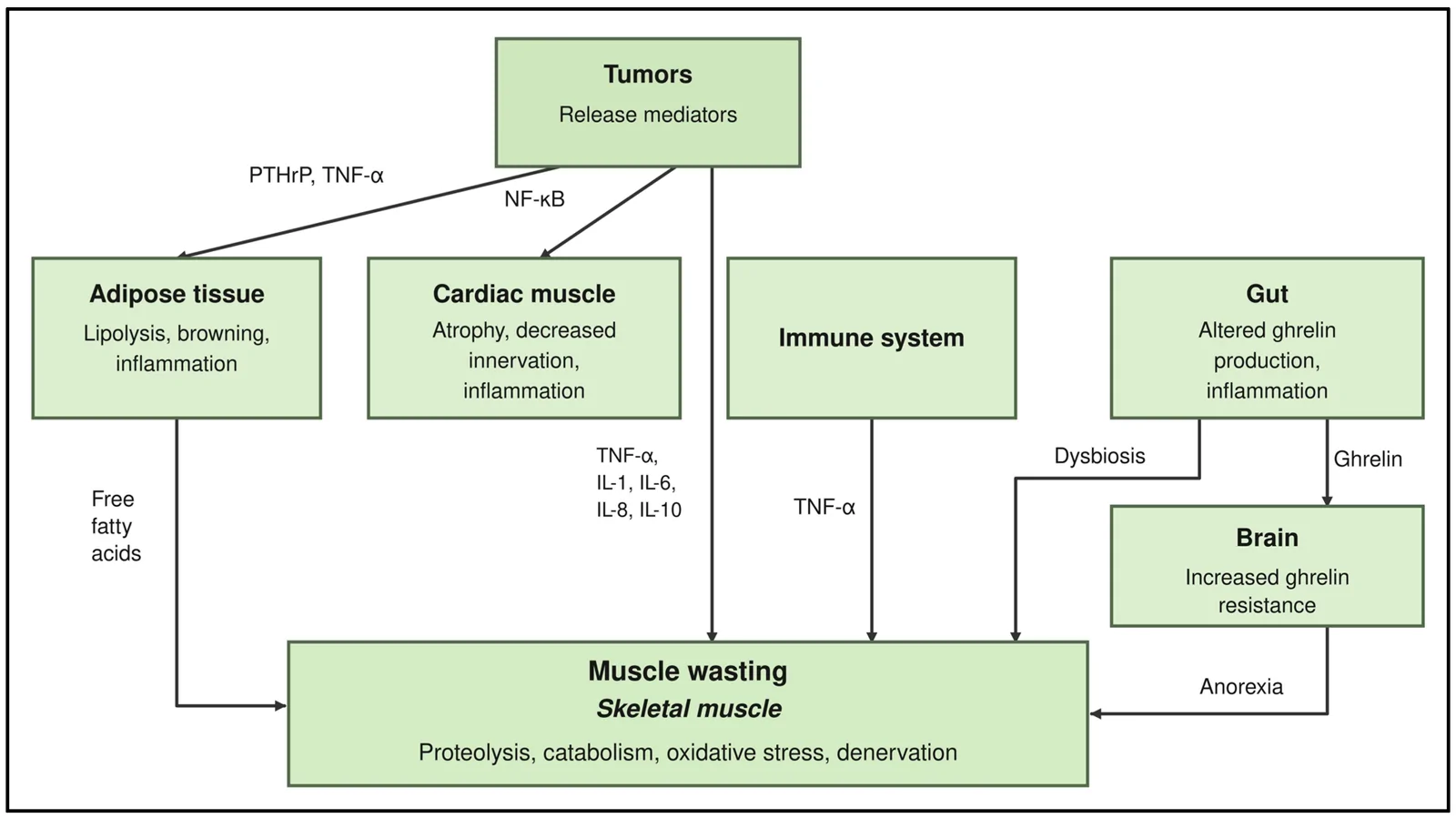
Inflammatory mediators and multi-organ involvement in cancer cachexia (adapted from Setiawan et al. 2023) [21]. Abbreviations: IL-1: interleukin-1; IL-6: interleukin-6; IL-8: interleukin-8; IL-10: interleukin-10; NF-κB: nuclear factor kappa B; PTHrP: parathyroid hormone-related protein; TNF-α: tumor necrosis factor alpha.

Research studies suggest that cancer cachexia is associated with circulating stable molecules. Further studies have indicated that these factors are cytokines [16]. Identifying inflammatory cytokines that drive tissue wasting is crucial for understanding cancer cachexia [17]. Tumor necrosis factor alpha (TNF-α) is recognized as a cytokine that can trigger metabolic shifts associated with cachexia. Due to its role in causing weight loss and muscle wasting in experimental models, TNF-α was initially referred to as cachectin [9]. Notably, cachectin/tumor necrosis has been demonstrated to represent two sides of an experimental coin [18]. TNF-α activates inflammatory signaling pathways, including nuclear factor kappa B, which promotes proteolysis in skeletal muscle while suppressing protein synthesis [19]. These mechanisms play a direct role in causing muscle wasting and disrupting metabolic balance. Cytokines like interleukin-1 (IL-1), interleukin-6 (IL-6), and leukemia inhibitory factor (LIF) are involved in the development of cachexia. High levels of these inflammatory mediators trigger catabolic signaling pathways and interfere with metabolic stability. Notably, IL-6 has been identified as a crucial factor in cancer cachexia, activating JAK-STAT signaling pathways that lead to muscle protein breakdown and suppress anabolic processes [20] (Figure 1). Chronic systemic inflammation induced by these cytokines also affects adipose tissue metabolism by stimulating lipolysis and reducing lipid storage capacity. These alterations further contribute to the progressive loss of body mass observed in cachectic patients [14] (Table 1).

### 2.5. Recognition of Multi-Organ Interactions

Studies have demonstrated that tumor-derived factors and inflammatory mediators influence skeletal muscle, adipose tissue, the liver, and the central nervous system. Skeletal muscle is the primary site of tissue loss in cachexia. The activation of the ubiquitin–proteasome system and other proteolytic pathways results in the progressive degradation of muscle proteins and a decline in muscle function [38]. Adipose tissue experiences notable metabolic changes, with heightened lipolysis and decreased adipogenesis leading to fat store depletion and shifts in systemic energy metabolism [15]. The liver contributes to the progression of cachexia by increasing the production of acute-phase proteins in response to systemic inflammation. This hepatic response elevates metabolic demand and enhances inflammatory signaling throughout the body [39]. Anorexia is another clinical sign of cancer cachexia [40]. Inflammatory cytokines impact hypothalamic pathways responsible for regulating appetite and energy homeostasis. These alterations frequently lead to anorexia and decreased caloric intake, thereby contributing to weight loss [41].

### 2.6. Immune Dysregulation and Inflammatory Signaling

**Figure 2:**
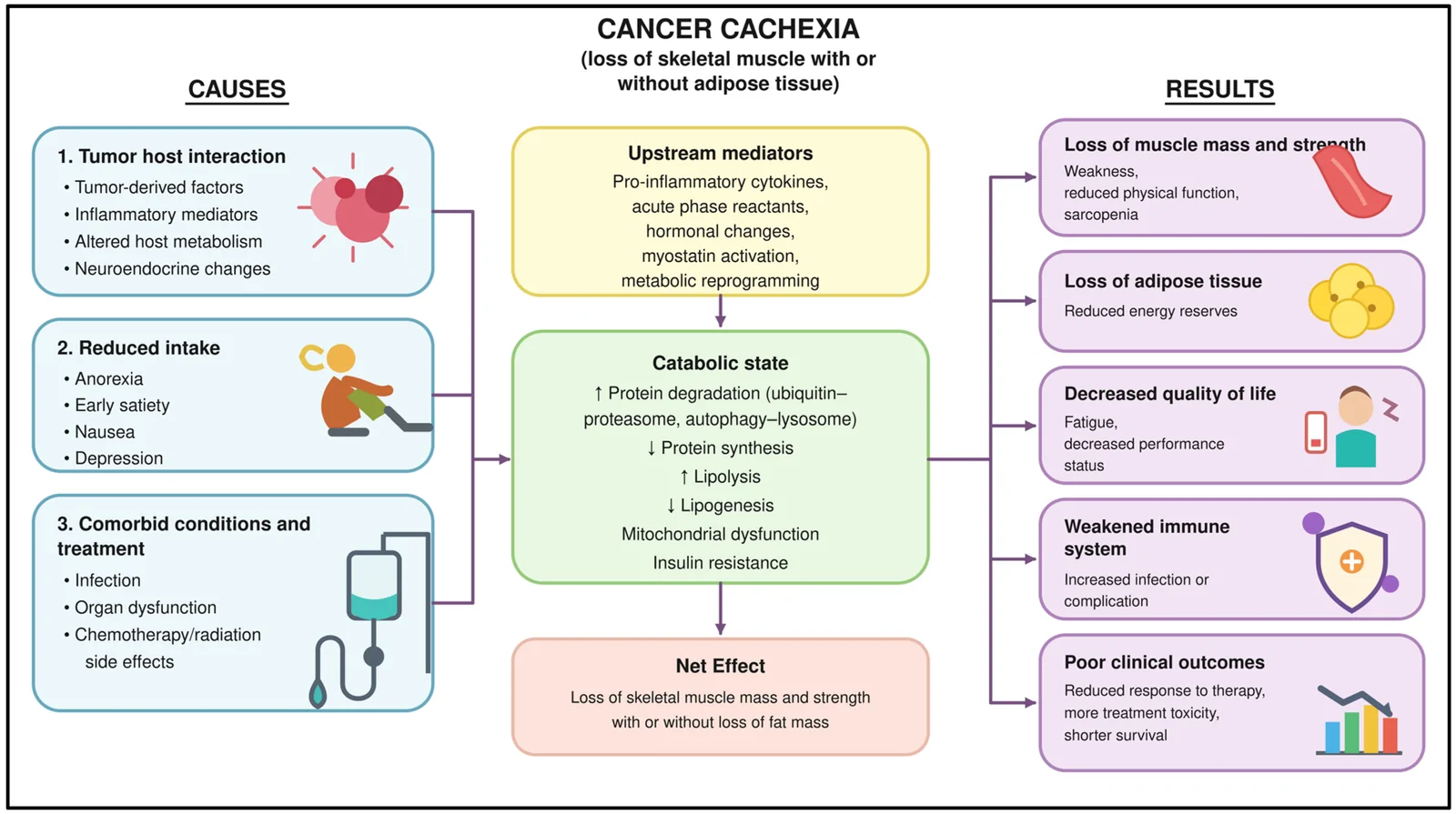
Causes and outcomes of cancer cachexia [47].

Immune dysregulation plays a key role in cancer cachexia. Tumor cells interact with immune cells to produce inflammatory cytokines and signaling molecules that disrupt metabolic homeostasis throughout the body. Persistent activation of inflammatory pathways leads to chronic catabolism and progressive tissue wasting. These immune-mediated mechanisms are crucial for cachexia pathogenesis [6,42,43]. The loss of skeletal muscle during cachexia may be attributed to mitochondrial dysfunction and compromised muscle regeneration. Mitochondrial changes diminish energy production and elevate oxidative stress within muscle cells, thereby fostering muscle degradation [44]. The skeletal muscle microenvironment is a coordinated network of myofibers, extracellular matrix, fibroblasts, vasculature, satellite cells, and resident and infiltrating immune cells. Cancer can disrupt myofiber homeostasis indirectly by altering these interacting compartments—yielding fibrosis, impaired regeneration, and reduced specific force—which aligns with the severity of weight loss even when mass is comparatively preserved. Current treatments have been unable to improve muscle function; therefore, consideration of the multiple cell types of the microenvironment is vital [45]. Signaling pathways involving myostatin and activin have been implicated in the regulation of muscle mass during cancer cachexia. Activation of these pathways inhibits muscle growth and promotes muscle atrophy [46] (Figure 2).

### 2.7. Systems Biology and Emerging Research Directions

Advances in systems biology have facilitated a deeper understanding of cancer cachexia as an intricate network of interactions among multiple organs and signaling pathways. Communication between skeletal muscle, adipose tissue, liver, immune cells, and the central nervous system plays a role in the development and maintenance of the cachectic state [48]. The gut microbiome modulates inflammation and metabolic dysfunction in cancer cachexia. Alterations in microbial composition may influence immune responses and metabolic regulation, thereby contributing to the progression of the syndrome [49]. Gut dysbiosis contributes to cachexia through reduced microbial diversity and loss of short-chain fatty acid producers, which weaken the intestinal barrier, allowing bacterial products to translocate into circulation and drive the systemic inflammation and muscle wasting that characterize the syndrome. Microbiota-targeted interventions, including prebiotics and dietary fibers, traditional and next-generation probiotics, and fecal microbiota transplantation, are being actively explored, though the clinical evidence to date remains limited [50]. Contemporary molecular methodologies have empowered researchers to detect alterations in gene expression, metabolic profiles, and signaling pathways associated with the progression of cachexia. These insights facilitate the identification of potential biomarkers and therapeutic targets for this condition [51].

### 2.8. Recent Advances and Therapeutic Strategies

Recent clinical research has emphasized the importance of early diagnosis and multimodal treatment approaches for cancer cachexia. Anti-inflammatory agents, anabolic therapies, appetite stimulants, and metabolic modulators targeting specific molecular pathways involved in muscle wasting are current areas of research [52]. Advances in biomarker discovery and metabolic profiling may allow earlier detection of cachexia during cancer progression. Early identification of patients at risk may improve treatment outcomes and quality of life [21]. Therapeutic strategies targeting inflammatory pathways, mitochondrial dysfunction, and muscle regeneration mechanisms involved in cachexia are now emerging [53,54].

Further research continues to explore the role of immune-mediated mechanisms and systemic inflammation in cancer-associated wasting [43]. In addition, studies investigating sarcopenic obesity have revealed complex interactions between adipose tissue metabolism and muscle loss in cancer patients [48]. Integrating immunology, molecular biology, metabolic science, and clinical research to develop effective therapeutic strategies for cachexia is essential [6,55].

### 2.9. Contemporary Perspective

Today, cancer cachexia is acknowledged as a complex systemic syndrome characterized by metabolic disturbances, ongoing inflammation, immune disorder, and communication across multiple organs. Over the years, research has shifted the view of this condition from a basic nutritional problem to a complex metabolic disease. Despite substantial progress in understanding its mechanisms, effective treatments are still scarce. Ongoing investigation into the molecular pathways responsible for muscle loss and metabolic issues is crucial for creating targeted therapies that could improve patient outcomes [55].

The potential targets of immunomodulation are vast, in line with the components of the immune system (spanning genes, molecules, cells, and organs), necessitating multiple routes for intervention, in addition to neutralization of individual cytokines. In addition to blocking pro-inflammatory mediators such as TNF-α and IL-6 with monoclonal antibodies, these mechanisms include enhancing the capacity of T cells to recognize tumor cells, utilizing the paracrine anti-inflammatory activity and macrophage- and T-cell-polarizing effects of mesenchymal stem cells, inhibiting intracellular signaling pathways (via JAK, calcineurin, mTOR, or IRAK4 inhibitors), and modulating inflammatory function. These approaches can be selective, ranging from broadly acting agents such as corticosteroids that affect all cell types to genomic strategies like RNA interference and CRISPR–Cas9 that can selectively target virtually any cellular protein, including transcription factors [56]. As cancer cachexia is not the result of a single mediator but a self-reinforcing network of tumor- and host-derived inflammatory signals, this explains why agents targeting one cytokine have shown limited benefit. This highlights the role of immunomodulatory strategies in restoring immune balance across cell populations rather than blocking a lone downstream signal, thereby addressing the underlying drivers of muscle wasting and treatment intolerance [57].

## 3. Drug Development in Cancer Cachexia

Early therapeutic strategies for cancer cachexia primarily concentrated on nutritional supplementation and appetite stimulation, adhering to the belief that cachexia was predominantly a result of decreased caloric intake and anorexia in patients with advanced malignancies [44]. Nutritional interventions were thus extensively used to restore body weight and energy balance in cachectic patients [58]. Megestrol acetate, a synthetic progesterone derivative, was one of the most extensively researched drugs for stimulating appetite in cancer cachexia. This drug has been found to stimulate appetite and increase caloric intake by modulating hypothalamic appetite-regulating pathways and suppressing pro-inflammatory cytokines. A study suggests that megestrol acetate causes weight gain in patients with advanced cancer [59]. Nevertheless, the augmentation in body weight associated with megestrol acetate therapy predominantly resulted from an increase in adipose tissue and fluid retention, rather than the restoration of lean skeletal muscle mass [44].

Corticosteroids were also frequently used to stimulate appetite and improve a sense of well-being in cancer patients experiencing cachexia [58]. Corticosteroids may provide short-term improvements in appetite and fatigue; however, their long-term use is limited by adverse effects such as muscle weakness, immunosuppression, and metabolic complications [58]. Cannabinoid-based therapies such as dronabinol were evaluated for appetite stimulation in cancer patients with cachexia. However, clinical trials evaluating cannabinoids demonstrated only modest improvements in appetite and limited benefits in terms of weight gain and muscle mass recovery [60]. These early pharmacological interventions highlighted the limitations of therapies focused on appetite stimulation and indicated that cancer cachexia involves complex metabolic disturbances that cannot be reversed by nutritional or appetite-based therapies alone [44].

As research advanced, an enhanced understanding of the biological mechanisms underlying cancer cachexia facilitated the development of therapies targeting specific molecular pathways involved in the syndrome. Cancer cachexia involves systemic inflammation driven by cytokines such as tumor necrosis factor-α, interleukin-1, and interleukin-6, which contribute to metabolic dysregulation and muscle wasting. Therefore, anti-inflammatory agents capable of suppressing cytokine signaling were explored as potential treatments for cachexia [61]. Thalidomide demonstrates the ability to inhibit tumor necrosis factor-α production and reduce inflammation in cancer patients with cachexia [58]. Clinical investigations suggested that thalidomide could improve appetite and body weight in certain cancer populations [58]. Monoclonal antibodies targeting the interleukin-6 receptor, including tocilizumab, have been investigated as potential treatments for cachexia-related inflammation [62]. Another IL-6 pathway inhibitor, ALD518, demonstrated promising results in early clinical trials, improving lean body mass and reducing fatigue in patients with advanced cancers [1]. These targeted approaches represented an important shift from purely symptomatic treatments toward therapies designed to interrupt the inflammatory mechanisms driving cancer cachexia [62].

More recent drug development efforts have increasingly focused on hormonal and metabolic regulators of appetite and muscle metabolism. One important therapeutic target is ghrelin, a peptide hormone that stimulates appetite and promotes anabolic signaling through growth hormone pathways. Ghrelin receptor agonists have therefore been investigated as treatments for cancer cachexia because of their ability to increase appetite and improve energy balance [2]. The orally active ghrelin receptor agonist anamorelin has been reported to increase appetite, body weight, and lean body mass in patients with cancer cachexia. Anamorelin has also been reported to improve symptoms such as fatigue and reduced food intake in cancer patients with advanced disease [63]. It has been approved in Japan for the treatment of cancer cachexia in certain malignancies, including lung cancer [2].

In addition to appetite-regulating therapies, drug development has also targeted pathways involved in skeletal muscle loss, particularly the myostatin and activin signaling pathways, which regulate muscle growth and degradation. Experimental therapies designed to inhibit myostatin or activin receptors have shown potential for increasing muscle mass in patients with cancer-related wasting [20]. Moreover, molecular targets such as growth differentiation factor-15 (GDF-15) are also being investigated for their potential role in modulating appetite and metabolic regulation in cancer cachexia [64]. Despite these advances, many investigators emphasize that cancer cachexia is a multifactorial syndrome and an effective treatment should comprise multimodal therapeutic strategies combining pharmacological agents, nutritional interventions, and physical rehabilitation [51].

Recent cancer cachexia drug development research shows a shift toward therapies that target the syndrome’s underlying biological mechanisms, though challenges in clinical trial design and regulatory approval persist. Studies now focus more on agents that influence metabolic and inflammatory pathways causing muscle wasting and systemic metabolic issues, rather than just addressing appetite or weight loss. For example, espindolol, a non-selective β-blocker, and monoclonal antibodies targeting growth differentiation factor-15 (GDF-15) are being explored. These interventions aim to directly target the molecular drivers of cachexia instead of merely alleviating its symptoms [65].

Ghrelin receptor agonists, such as anamorelin, have undergone comprehensive investigation in randomized controlled trials, including the phase III ROMANA 1 and ROMANA 2 studies. These trials demonstrated enhancements in lean body mass, body weight, and symptoms associated with anorexia in patients diagnosed with non-small cell lung cancer and cachexia [66]. Nonetheless, even with these positive metabolic results, gains in functional measures like hand-grip strength did not reach statistical significance, highlighting a major challenge in assessing clinical benefits in cachexia [65]. The selection of appropriate clinical endpoints continues to present challenges, as improvements in body composition do not invariably correspond to measurable enhancements in physical function or survival. This discrepancy complicates the regulatory evaluation of novel therapies [2].

Obtaining regulatory approval has been challenging for many investigational drugs, even when initial clinical results seem promising. For instance, the ghrelin agonist anamorelin has been approved in Japan for treating cancer cachexia but has not yet gained approval in Europe or North America. This delay is partly due to discrepancies between gains in lean body mass and the functional improvements that regulators require [2]. Likewise, other targeted therapies such as myostatin inhibitors, cytokine-targeting antibodies, and selective androgen receptor modulators have shown potential benefits in early-phase studies but have not yet achieved consistent success in late-phase trials. These challenges underscore the complexity of cancer cachexia and have led researchers to increasingly advocate for multimodal treatment strategies combining pharmacological therapies with nutritional and exercise interventions, while continuing to refine clinical trial endpoints and regulatory frameworks for this multifactorial syndrome [2].

## 4. Lessons from the Failure of Multiple Clinical Trials Targeting Single Pathways

The international consensus definition of cachexia as a “multifactorial syndrome characterized by ongoing loss of skeletal muscle mass (with or without fat loss) that cannot be fully reversed by conventional nutritional support and leads to progressive functional impairment” [4] explicitly highlights metabolic dysregulation, systemic inflammation, and tumor–host interactions as central drivers. However, many therapeutic strategies have focused on single mechanistic axes such as appetite stimulation (ghrelin agonists), anabolic signaling (androgens and selective androgen receptor modulators), or isolated cytokine blockade (TNF-α, IL-6, and IL-1) [17]. Cachexia involves complex crosstalk between tumor-derived factors, host inflammatory mediators, neuroendocrine changes, and altered muscle protein turnover, suggesting that reductionist approaches are unlikely to provide durable clinical benefit [48]. Similarly, the activation of proteolytic systems, such as the ubiquitin–proteasome pathway and autophagy, alongside anorexia and metabolic inefficiency, affects systemic pathophysiology [9].

High failure rates in phase II/III trials may be due to differences in drug mechanism and syndrome complexity. Agents targeting a single pathway may influence body composition but fail to meaningfully alter overall function, survival, food intake or quality of life because compensatory mechanisms and ongoing tumor-driven inflammation persist [48]. These findings highlight that cachexia is not merely a nutritional deficit or isolated endocrine abnormality but a multi-system metabolic challenge requiring integrated therapeutic strategies.

### 4.1. Case Studies

In the ROMANA 1 and 2 Phase III trials evaluating anamorelin in patients with advanced non-small cell lung cancer (NSCLC) and cachexia, anamorelin markedly increased lean body mass compared to placebo. However, hand-grip strength, an essential endpoint, did not show a significant improvement. Consequently, although appetite and certain patient-reported outcomes demonstrated enhancement, the lack of functional gains impeded regulatory approval. These findings thus reveal a dissociation between body composition and clinically meaningful function [67].

Similarly, anti-inflammatory strategies targeting individual cytokines have largely proven ineffective. Initial trials employing TNF-α inhibitors, such as etanercept, in cases of advanced cancer did not exhibit substantial enhancements in weight or survival [68]. Trials targeting IL-6 (e.g., clazakizumab or ALD518) showed modest improvements in anemia or fatigue but inconsistent effects on lean body mass or overall outcomes. IL-1 blockade (e.g., MABp1 targeting IL-1α) demonstrated some symptom improvement in colorectal cancer but failed to impact survival [69]. Therefore, these case studies demonstrate that targeting a single inflammatory mediator may be ineffective due to redundancy and compensatory signaling within the cytokine network.

### 4.2. Common Pitfalls

Methodological limitations may result in therapeutic failures. Inadequate patient stratification has affected observable effects. Cachexia occurs in a spectrum ranging from pre-cachexia to refractory cachexia, yet many trials enrolled heterogeneous populations without biomarker-driven selection [4]. Endpoint selection has often focused on weight gain or lean body mass rather than physical performance, functional capacity, or survival. The ROMANA trials highlighted the misalignment between increased lean mass and unchanged strength [67]. Moreover, Prado et al. (2018) emphasized that muscle quantity does not necessarily equate to muscle quality or performance [69]. Nonetheless, progressive malignancy itself influences outcomes [69]. Tumor burden, chemotherapy toxicity, and systemic inflammation independently drive muscle catabolism, making it difficult for anabolic agents to overcome ongoing catabolic stimuli [52]. Further, cancer trajectory and concurrent treatments may also affect the interpretability of results.

### 4.3. Broader Implications

These failures collectively highlight cancer cachexia as a multi-organ failure syndrome that affects skeletal muscle, adipose tissue, the liver (through the acute-phase response), the brain (regulating appetite), and the immune system. Central to this process is tumor–host interaction, which drives metabolic reprogramming and systemic inflammation. The continued presence of cachexia despite nutritional support and single-drug treatments indicates that addressing the root causes, such as systemic inflammation, tumor-derived factors, and immune disorders, may be crucial [5,6]. Multimodal therapy combining pharmacologic, nutritional, and exercise interventions appears to be essential for addressing cancer cachexia [48]. Thus, past failures highlight that simply targeting one pathway is inadequate because the syndrome is characterized by multiple factors and involves systemic dysregulation. Therefore, emerging strategies should focus on integrated immune–metabolic modulation rather than isolated appetite or anabolic stimulation (Table 2).

## 5. The Need for a Fundamental Change in Reversing the Syndrome

### 5.1. Critique of Current Paradigms

Cancer cachexia is characterized by ongoing skeletal muscle loss that cannot be fully reversed by conventional nutritional support and leads to progressive functional impairment. Cachexia is not only the result of reduced caloric intake but also of complex metabolic abnormalities and systemic inflammation [4]. Subsequent mechanistic analyses have demonstrated that tumor–host interactions activate a coordinated network involving skeletal muscle proteolysis, adipose tissue lipolysis, hepatic acute-phase response, neuroendocrine alterations, and immune dysregulation [5,6,48].

Systemic inflammation is a key factor, with pro-inflammatory cytokines IL-6, TNF-α, and IL-1β promoting muscle breakdown, loss of appetite, insulin resistance, and alterations in hepatic metabolism [9,20]. Cytokine signaling operates within redundant and compensatory immune networks, limiting the efficacy of single-cytokine blockade strategies [9]. Cachexia is therefore increasingly conceptualized as an immune–metabolic disorder, where immune disorder and metabolic reprogramming are tightly linked [6,52,67].

Clinical trial failures targeting isolated pathways such as appetite stimulation or muscle anabolism highlight differences between disease complexity and therapeutic design. Improvements in lean body mass frequently fail to translate into enhanced muscle strength, physical function, or survival [52,67]. Observations demonstrate that these approaches do not sufficiently address the interconnected inflammatory, metabolic, neuroendocrine, and immune disturbances underlying the syndrome.

### 5.2. Paradigm Shift

Given this complexity, most experts recommend a multimodal approach that targets the root causes for managing cachexia [4,48]. International consensus recommendations highlight the importance of integrating pharmacologic agents with nutritional intervention, exercise therapy, and anti-inflammatory strategies, rather than using monotherapy alone [70]. Ponsegromab, a GDF-15-neutralizing antibody, produced meaningful gains in body weight, appetite, and physical activity in GDF-15-elevated NSCLC, pancreatic, and colorectal cancer patients in both phase 1b and phase 2 (PROACC-1) trials. However, a survival benefit has not been demonstrated [71,72]. Therefore, single-pathway therapies are inherently limited, as GDF-15 is an upstream mediator whose downstream effects are restricted, acting via GFRAL/RET to drive anorexia, lipolysis, and muscle wasting through the HPA axis, Smad, PI3K/Akt, and NF-κB pathways. GDF-15-independent contributions of IL-6, TNF-α, TGF-β/myostatin, gut dysbiosis, and multi-organ crosstalk (gut–brain, gut–liver, gut–muscle, muscle–adipose, muscle–bone axes) that collectively drive the cachectic phenotype are crucial. Hence, blocking GDF-15 alone may not be enough [71].

Biomarker-guided patient stratification is increasingly viewed as essential. Inflammatory markers (e.g., CRP, IL-6), body composition analysis, and metabolic signatures may help identify subgroups most likely to benefit from specific interventions [48]. A systematic review of endpoints in cachexia trials highlighted substantial heterogeneity and emphasized the need for validated biomarker-based outcomes to enhance trial outcomes and interpretability [73].

The integration of cachexia management into routine oncology practice has also been recommended. Early screening and intervention, rather than late-stage reactive treatment, may enhance functional outcomes and treatment tolerance [74]. By aligning cachexia management with standard cancer care protocols, clinicians may mitigate treatment toxicity, enhance physical performance, and potentially influence overall prognosis [48,74]. Thus, the emerging paradigm supports combination therapy, biomarker-based personalization, and early integrated care, targeting the underlying inflammatory and metabolic drivers rather than isolated downstream manifestations.

### 5.3. Regulatory and Methodological Reforms

Reforming clinical trial design is essential for progressing therapeutic development. Previous studies focused on endpoints including body weight or lean body mass, but these measures do not reliably correlate with functional improvements [67,73]. Regulatory requirements now focus on patient-centered endpoints such as hand-grip strength, physical performance tests, and quality-of-life measures [67]. Adaptive and biomarker-stratified trial designs may improve sensitivity to treatment effects in heterogeneous populations. Incorporating composite endpoints that combine body composition, functional performance, and inflammatory biomarkers may provide a clinically meaningful benefit. Cachexia is comparable to aging-related sarcopenia and chronic inflammatory diseases [52,69]. Collaborative, interdisciplinary frameworks involving oncology, geriatrics, immunology, and nutrition science are therefore essential to address shared pathophysiology and enhance generalizability. Recognizing cachexia as a systemic comorbidity rather than a secondary symptom of advanced cancer may facilitate more appropriate regulatory evaluation and therapeutic prioritization [74]. Therefore, these changes indicate a need to shift toward integrated, biologically informed, and patient-centered research frameworks aligned with the systemic nature of cancer cachexia.

## 6. Emerging and Promising Immunomodulatory Approaches

Cancer cachexia is driven primarily by systemic immune dysregulation rather than nutritional deficiency, making immunomodulation a rational therapeutic strategy. Chronic inflammation mediated by cytokines such as IL-6, TNF-α, and IL-1β induces muscle wasting and metabolic dysfunction via pathways like JAK/STAT and NF-κB [6,75]. The tumor microenvironment further amplifies this process through dynamic interactions between tumor and immune cells, sustaining cytokine signaling and immune suppression [76]. Tumor-induced immune disorder, characterized by T-cell dysfunction, lymphopenia, and expansion of MDSCs, creates a catabolic state linked to poor outcomes [6]. Similar cytokine-driven immune imbalance is observed in chronic inflammatory conditions, reinforcing the centrality of immune dysregulation in disease progression [77]. While early approaches targeting single mediators (e.g., GDF-15 or individual cytokines) show partial benefit, they are limited by pathway redundancy, compensatory mechanisms, and patient heterogeneity [6]. Thus, broader immunomodulatory strategies that restore immune homeostasis are essential for meaningful reversal of cachexia.

Emerging therapies increasingly target the tumor–immune–host axis in an integrated manner. Cytokines regulate tumor sensitivity and immune responses, influencing apoptosis, immune activation, and therapeutic susceptibility [76]. Advances in cytokine engineering, biologics, and JAK inhibitors highlight the importance of modulating multiple inflammatory pathways simultaneously [77]. Chronic inflammation within the tumor microenvironment promotes immune loss and treatment resistance, necessitating systemic immune reprogramming rather than isolated cytokine blockade [75]. Spatial and metabolic heterogeneity complicates targeted approaches, reinforcing the need for multi-pathway interventions [76]. Consequently, modern strategies focus on restoring immune balance while enhancing anti-tumor immunity. This represents a shift from single-target inhibition to systems-level immunomodulation.

R-ketorolac is a promising next-generation immunomodulator targeting tumor-induced immune disorders. Preclinical studies demonstrate the reversal of cachexia in tumor-bearing models, including reduced weight loss and muscle wasting independent of tumor size or food intake. Treatment restores immune balance by improving T-cell populations and reducing inflammatory cytokines such as IL-6. These effects translate into improved survival and near-complete reversal of cachexia phenotypes in animal models. Its mechanism suggests root-cause targeting of immune dysfunction rather than symptomatic relief. Given the multifactorial nature of cachexia, combination strategies with immunotherapy or chemotherapy may further enhance outcomes. R-ketorolac is a strong candidate within multimodal, immune-centric treatment paradigms [6]. However, in contrast to other conventional NSAIDs, it acts independently of cyclooxygenase inhibition and of the Rac1 inhibition associated with its anti-tumor effects [78]. Moreover, clinical translation of R-ketorolac will require evaluation of safety, pharmacokinetics, and target engagement. Potential biomarkers include CRP, IL-6, and immune-cell profiles, while patients with early or progressive cachexia and systemic inflammation may be appropriate candidates. Early trials should assess functional and patient-reported outcomes alongside body composition and inflammatory biomarkers [79,80].

Currently, clinicians can provide nutritional monitoring and support patients right from cancer diagnosis, as reduced intake from anorexia, nausea, and mucositis is an early and modifiable feature of the syndrome. They can profile every patient’s cachexia stage using established criteria such as degree of weight loss, BMI, and systemic inflammation [81] to guide a combined intervention that pairs nutritional support with exercise and rehabilitation [1] and pharmacological treatment, rather than relying on any single agent [70].

## 7. Conclusions

Cancer cachexia is now clearly identified as a complex immune–metabolic syndrome in which tumor–host interactions, systemic inflammation, and multi-organ dysfunction converge to drive progressive decline, rendering traditional single-pathway and symptom-focused interventions inadequate. The recurrent lack of success in such approaches has elucidated a vital insight: substantial clinical benefit will not be achieved by targeting isolated mechanisms but rather by addressing the integrated biological network that underpins the syndrome. Emerging evidence highlights immunomodulation, especially approaches that restore systemic immune balance, as a promising, mechanism-based direction. Early results with agents like R-ketorolac suggest the possibility of addressing cachexia at its core. Future efforts should adopt a framework that combines multimodal, biomarker-guided, and patient-centered strategies, integrating immunotherapy, metabolic regulation, nutrition, and physical rehabilitation into routine cancer care. Advances in systems biology, clinical trial design, and translational science offer a real and promising chance to reframe cancer cachexia from an unavoidable outcome of advanced disease into a treatable condition, ultimately enhancing treatment tolerance, functional ability, and survival for cancer patients.

## Figures and Tables

**Table 1:** Cytokines and their role in cachexia.

Cytokine	Source Cells	Cancer-Affected Organs	Experimental Model	Effects Observed in Cachexia	References
IL-1α	Macrophages and endothelial cells	Pancreas, breast, colorectal regions	Rodents (mice and rats)	Enhanced lipid breakdown, reduced appetite, body weight decline, decreased hunger signals	[22,23,24]
IL-1β	Macrophages	Stomach, breast, lung, and multiple other sites	Mice and humans	Appetite loss, reduction in body mass, sarcopenia, fatigue, disrupted mitochondrial function	[25,26,27]
IL-6	Activated macrophages	Colorectal region, pancreas, and additional organs	Mice and humans	Fat tissue depletion, skeletal muscle degradation, impact on gut and liver, mitochondrial dysfunction	[23,24,27]
TNF-α	Activated macrophages, CD4+ cells, neutrophils, mast cells, eosinophils, and neurons	Pancreas, lungs, colon, and other organs	Mice and humans	Appetite suppression, loss of muscle and fat mass, insulin resistance, elevated energy usage, mitochondrial impairment	[28,29,30]
GDF-15	Adipocytes, macrophages, endothelial cells, vascular smooth muscle cells, cardiomyocytes, and trophoblastic cells	Lungs, pancreas, colorectal region, prostate, and others	Mice	Altered energy regulation, appetite suppression, muscle wasting, fat depletion, reduced bone density, anemia	[24,31,32]
IFNγ	Activated T cells and NK cells	Pancreas, lung, colon, prostate	Rodents (mice and rats)	Decrease in body weight, lowered appetite, fat tissue atrophy	[24,33,34]
LIF	Melanoma cells, neuroepithelioma cells, gastric cancer cells, pancreatic cancer cells,	Epididymal fat, spleen, and different muscle types	Mice	Loss of body fat, myotube atrophy	[35,36,37]

Abbreviations: CD4+ cells: cluster of differentiation 4-positive T cells; GDF-15: growth differentiation factor 15; IFN-γ: interferon gamma; IL-1α: interleukin-1 alpha; IL-1β: interleukin-1 beta; IL-6: interleukin-6; LIF: leukemia inhibitory factor; NK cells: natural killer cells; TNF-α: tumor necrosis factor alpha.

**Table 2:** Overview of pharmacotherapies targeting cancer cachexia pathways.

Drug	Class	Mechanism of Action	References
Anamorelin	Ghrelin receptor agonist (orally active)	Stimulates appetite and anabolic signaling via the growth hormone pathway; increases appetite, body weight, and lean body mass	[63,66,67]
Clazakizumab (formerly ALD518)	IL-6 pathway inhibitor/monoclonal antibody	Blocks IL-6 signaling; reported improved lean body mass and reduced fatigue; modest effect on anemia/fatigue; inconsistent effect on lean mass across trials	[69]
Corticosteroids	-	Stimulates appetite and sense of well-being (short-term; limited by muscle weakness and immunosuppression)	[58]
Dronabinol	Cannabinoid	Appetite stimulation (modest effect on appetite/weight/muscle mass)	[60]
Espindolol	Non-selective β-blocker	Targets molecular/metabolic drivers of cachexia (beyond symptomatic relief)	[65]
Etanercept	TNF-α inhibitor	Blocks TNF-α signaling (no substantial improvement in weight/survival)	[68]
GDF-15-targeting monoclonal antibodies	Monoclonal antibody	Modulates GDF-15 signaling to affect appetite and metabolic regulation	[64,65]
MABp1	IL-1α-blocking antibody	Blocks IL-1α signaling; some symptom improvement but no survival benefit	[69]
Megestrol acetate	Synthetic progesterone derivative	Modulates hypothalamic appetite-regulating pathways; suppresses pro-inflammatory cytokines (weight gain mainly from fat/fluid, not lean mass)	[44,59]
Myostatin/activin receptor inhibitors	Anabolic pathway inhibitor	Blocks myostatin/activin signaling to increase muscle mass	[20]
R-ketorolac	Next-generation immunomodulator	Restores immune balance (improves T-cell populations, reduces IL-6 levels); reverses cachexia independent of tumor size or food intake	[6]
Thalidomide	Anti-inflammatory/immunomodulatory	Inhibits TNF-α production; reduces inflammation	[58]
Tocilizumab	Monoclonal antibody	Targets IL-6 receptor, suppressing IL-6-mediated inflammation	[62]

Abbreviations: GDF-15: growth differentiation factor 15; IL-1α: interleukin-1 alpha; IL-6: interleukin-6; MABp1: monoclonal antibody p1; TNF-α: tumor necrosis factor alpha.

## Data Availability

No new data were created or analyzed in this study. Data sharing is not applicable to this article.
